# Encapsulation of *Bifidobacterium pseudocatenulatum* Strain G4 within Bovine Gelatin-Genipin-Sodium Alginate Combinations: Optimisation Approach Using Face Central Composition Design-Response Surface Methodology (FCCD-RSM)

**DOI:** 10.1155/2019/4208986

**Published:** 2019-04-10

**Authors:** Khalilah Abdul Khalil, Suhaimi Mustafa, Rosfarizan Mohammad, Arbakariya Bin Ariff, Siti Aqlima Ahmad, Farrah Aini Dahalan, Mohd Yazid Abdul Manap

**Affiliations:** ^1^Department of Biomolecular Science, School of Biology, Faculty of Applied Sciences, Universiti Teknologi MARA, Sec. 2, 40150 Shah Alam, Selangor, Malaysia; ^2^Halal Products Research Institute, Universiti Putra Malaysia, Putra Infoport, 43400 Serdang, Selangor, Malaysia; ^3^Faculty of Biotechnology and Biomolecular Sciences, Universiti Putra Malaysia, 43400 Serdang, Selangor, Malaysia; ^4^The School of Environmental Engineering, Universiti Malaysia Perlis, Kompleks Pengajian Jejawi 3, 02600 Arau, Perlis, Malaysia; ^5^Faculty of Food Science and Technology, Universiti Putra Malaysia, 43400 Serdang, Selangor, Malaysia

## Abstract

Bovine gelatin is a biopolymer which has good potential to be used in encapsulating matrices for probiotic candidate *Bifidobacterium pseudocatenulatum* strain G4 (G4) because of its amphoteric nature characteristic. Beads were prepared by the extrusion method using genipin and sodium alginate as a cross-linking agent. The optimisation of bovine gelatin-genipin-sodium alginate combinations was carried out using face central composition design (FCCD) to investigate G4 beads' strength, before and after exposed to simulated gastric (SGF), intestinal fluids (SIF), and encapsulation yield. A result of ANOVA and the polynomial regression model revealed the combinations of all three factors have a significant effect (*p* < 0.05) on the bead strength. Meanwhile, for G4 encapsulation yield, only genipin showed less significant effect on the response. However, the use of this matrix remained due to the intermolecular cross-linking ability with bovine gelatin. Optimum compositions of bovine gelatin-genipin-sodium alginate were obtained at 11.21% (w/v), 1.96 mM, and 2.60% (w/v), respectively. A model was validated for accurate prediction of the response and showed no significant difference (*p* > 0.05) with experimental values.

## 1. Introduction

Probiotics such as bifidobacteria are microorganisms that confer health benefits upon application in sufficiently high amount of viable cells [1]. Upon consumption, the viability of probiotic in a product is an important consideration for their efficacy as they must be metabolically stable and active in the product as well as during the passage through the stomach. In addition, they have beneficial effects when they are in the intestine of the host [2]. The consumption of probiotic at a level between 10^8^ and 10^9^ cfug^−1^ per day is commonly recommended for adequate probiotic consumption [3, 4]. However, there are still limitations with respect to the low viability of probiotic in products and upon reaching the target area. *B. pseudocatenulatum* was first isolated and identified by Shuhaimi et al. [5] using the randomly amplified polymorphic DNA (RAPD) analysis approach. The characteristics of the isolates as probiotic candidate were evaluated by investigating their acid and bile tolerance abilities. It was proven that, among the *B. pseudocatenulatum* isolates, G4 strain demonstrated the best strain that was tolerant to low pH [6] and high bile concentration, which was up to 4% [7]. Moreover, the safety evaluations study of the strain also suggested the suitability of the strain as a probiotic candidate [8].

A few approaches to increase the resistance of these sensitive microorganisms against adverse external conditions are currently receiving considerable interest. Encapsulation is currently introduced to protect bacteria against severe environmental factors [9, 10]. The intention of encapsulation is to create a microenvironment in which bacteria will survive during the processing stage and storage. In addition, the bacteria will be able to release activity of the cells at the appropriate site in the digestive tract.

Among the encapsulation procedures, cells encapsulated in gelled biopolymer of calcium-alginate matrix is commonly used because of its simplicity, nontoxicity, biocompatibility, and low cost [11, 12]. However, alginate containing lactic acid bacteria tends to be liquefied by lactic acid and high in porosity [13]. Gelatin is reported as an excellent candidate to be incorporated with alginate due to its amphoteric nature [14]. These hydrocolloids are miscible at pH > 6 because both polymers carry negative charges and repel one another. However, when the pH is below its isoelectric point, the net charge of gelatin becomes positive and causes a strong interaction with the negatively charged biopolymers [9]. Annan et al. [15] reported that the combination of alginate-coated gelatin-genipin to form microspheres using the emulsion method provides a significant protection for *B. adolescentis* from harsh acidic condition of simulated gastric juice. However, the previous studies are limited to porcine gelatin application only. Nevertheless, Islam forbids the consumption of any pork-related products. As a result, an alternative source of animal gelatin like bovine gelatin needs to be explored for its ability to encapsulate potential probiotic *B. pseudocatenulatum* strain G4 (G4). This is an important approach to protect the cells from harsh acidic conditions of simulated gastric juice and deliver the cells to the target site. Genipin, a new cross-linker derived from *Gardenia jasminoides* plant, was used with the purpose to cross-link with gelatin to increase the gelatin's mechanical and thermal stability [16]. The optimal ratio of bovine gelatin, genipin, and alginate needs to be determined due to the difference in bloom strength of gelatin used in this study.

Therefore, the objectives of this study were (1) to optimize formulation of bovine gelatin, genipin, and alginate as encapsulating matrices using response surface methodology (RSM), (2) to understand the interaction effect of all three factors (bovine gelatin-genipin-sodium alginate) on the bead strength stability (before and after exposed to simulated gastric fluid (SGF)/ simulated intestinal fluid (SIF)), and (3) to investigate the encapsulation yield of G4.

## 2. Materials and Methods

### 2.1. *B. pseudocatenulatum* Strain G4 Preparation

Microorganism used in this study was G4 which was obtained from the Probiotic Laboratory, Faculty of Food Science and Technology, Universiti Putra Malaysia. It was previously isolated from breast-fed infant feces [5, 17]. The strains used were stored at −20°C in a mixture of glycerol: TPY broth (trypticase phytone yeast extract medium: Scharlau-Chemie, Barcelona, Spain), at a ratio of 20 : 80. Prior to encapsulation, the strain was initially activated in previously optimised skim milk medium supplemented with yeast extract formulation [18]. G4 was then transferred into a 2 L stirred tank bioreactor (BIOSTAT® B, B. Braun Biotech International, Melsungen, Germany) for further cultivation to obtain a cell density of about 10^10^–10^11^ cfu·mL^−1^.

### 2.2. Encapsulation of Strain G4

Encapsulation of approximately 10^10^ cfu·mL^−1^ of strain G4 was prepared using the extrusion method as described by Khalilah et al. [19]. One percent (v/v) of washed cell suspension was added to sodium alginate (Sigma Aldrich Co, St Louis, MO, USA) to obtain a cell–to-sodium alginate ratio of 1 : 10. Aqueous bovine gelatin (type B: 75 bloom strength) was prepared separately by dissolving the gelatin powder in distilled deionized water (DDW). An internal coating was obtained by adding the cell-alginate mixture into each aqueous gelatin and left to stir for 30 min. Genipin (Challenge Bioproducts Co, Taiwan, PRC) was subsequently added into the mixture and left to stir at a constant speed (200 rpm) for 60 min using a Digital Stirrer (WiseStir®, MSH-30D, Korea). The mixture was then injected through a syringe needle (23G) into hardening solution, 0.1 M CaCl_2_, by using a peristaltic pump. The distance between the syringe and CaCl_2_ (Fluka, St. Louis, USA) was 10 cm, and the dropping rate was 159 drops·min^−1^. The beads were harvested, filtered, and washed twice with deionized water before being transferred to sterile SCHOTT DURAN® laboratory glass bottles with screw caps (SCHOTT DURAN®, Mainz, Germany) containing 0.1% (w/v) sterile peptone solution and kept at 4°C for further analysis. All glassware and solutions including sodium alginate, genipin, gelatin, CaCl_2_, and peptone solution were autoclaved at 121°C for 15 minutes before use.

### 2.3. Preparation of Simulated Gastric Fluid (SGF) and Simulated Intestinal Fluid (SIF)

SGF and SIF were prepared based on Gildas et al. [20], with modification. SGF was prepared by adding 9 g·L^−1^ of sodium chloride (Merck, Darmstadt, Germany) and 3 g·L^−1^ pepsin from porcine stomach mucosa (Sigma Aldrich Co, St Louis, MO, USA), which was then adjusted to pH 1.2 with 1 M hydrochloride acid. The mixture was sterile filtered through a 0.45 *μ*m nylon membrane. Meanwhile, SIF was prepared by adding 9 g·L^−1^ of sodium chloride (Merck, Darmstadt, Germany), 10 g·L^−1^ trypsine from porcine pancreas (Sigma Aldrich Chemie, Steinem, Switzerland), and 3 g·L^−1^ of bile salts (Fluka, St. Louis, USA), with its pH adjusted to 6.8 with 1 M sodium hydroxide. Both fluids were sterile filtered through a 0.45 *μ*m membrane. Washed beads with entrapped G4 were added to 5 mL of each fluids consequently and incubated for 2 h at 37°C. The bead strengths were determined after the incubation period.

### 2.4. Analyses

#### 2.4.1. Enumeration of Encapsulation Yield (EY%)

Phosphate buffer at 0.05 M and pH 8.0 was used to release the entrapped strain G4 in alginate-gelatin-genipin matrices because phosphate ions chelate calcium, thereby weakening the alginate for effective release of cells. The homogenized sample was diluted to appropriate concentration and spread on TPY agar (Scharlau-Chemie, Barcelona, Spain). The plates were subsequently incubated anaerobically using Anaerocult ®A (Merck, Darmstadt, Germany) for 48 h to 72 h at 37°C. The encapsulated cells were enumerated as log 10 cfu·mL^−1^. The encapsulation yield (EY), which is a combined measurement of the efficacy of entrapment and survival of viable cells during the encapsulation procedure, was calculated as follows:

(1) $$\begin{array}{c} \text{EY}=\left(\frac{N}{N_{0}}\right)\times 100, \end{array}$$

where *N* is the number of viable entrapped cells released from the beads and *N*_0_ is the number of free cells added to the biopolymer mix during the encapsulation procedure.

#### 2.4.2. Bead Strength Determination

The method for determining the strength (g) of the beads was modified from a previous report by Edward-Lévy and Lévy [21]. An analysis of the mechanical property of the beads was carried using a texture analyzer (TA.HD plus, Stable Micro System, UK) equipped with a 50 kg load cell and a cylindrical aluminum probe of 36 mm in diameter. The probe was positioned to touch the beads, recorded as the initial position, and then the probe flattened the beads. The compression of the beads was measured using the following conditions: test mode: hardness (g), pretest speed: 1 mm·s^−1^, test speed: 2 mm·s^−1^, target mode: strain, distance: 5 mm, trigger force: 50 g, and time: 5 sec. The probe was removed when the beads was reduced to 50% of its original height. The maximum force (g) at 50% displacement represents the strength of the beads was recorded and analyzed by Texture Exponent 32 software program (version 3.0). Three measurements were performed on each sample.

#### 2.4.3. Experimental Design and Statistical Analysis

*(1) Screening*. Appropriate ranges of bovine gelatin, sodium alginate, and genipin were determined by the screening experiment, which were 13% (w/v) for bovine gelatin, 1–5% (w/v) for sodium alginate, and 0, 5, 10, 15, 20, and 50 mM (at final concentration) for genipin. Experiments are carried out in triplicates.

*(2) Optimisation*. Response surface methodology-face-centered composite design (RSM-FCCD) was used to design experiments, model, and optimize four response variables, namely, encapsulation yield (%), bead strength (g), bead strength after SGF (g), and bead strength after SIF (g). Each independent variable was coded at three levels between −1 and +1, where the variables bovine gelatin, sodium alginate, and genipin were changed in the ranges shown in Table 1. The ranges of selected parameters were determined by preliminary experiments. Fourteen experiments were augmented with five replications at the centre points to evaluate the pure error and to fit a quadratic model. The optimum growth point predicted by the quadratic model was expressed as follows:

(2) $$\begin{array}{c} y=\beta_{0}+\sum \beta_{1}x_{1}+\sum \beta_{2}x_{2}+\sum \beta_{3}x_{3}+\sum \beta_{11}x_{1}^{2}+\sum \beta_{22}x_{2}^{2}+\sum \beta_{33}x_{3}^{2}+\sum \beta_{12}x_{1}x_{2}+\sum \beta_{13}x_{1}x_{3}+\sum \beta_{23}x_{2}x_{3}. \end{array}$$

*(3) Verification*. Verification was carried out by conducting an experiment based on optimal encapsulating matrices setting through a mathematical model generated from RSM-FCCD. The statistical software package Design-Expert version 6.0.6 (Stat-Ease Inc., Minneapolis, USA) was used for regression analysis of experimental data and to plot response surface.

## 3. Results and Discussion

### 3.1. Screening of Encapsulating Matrices

Conventional screening was carried out to determine the range of encapsulating matrices composition using bovine gelatin, genipin, and sodium alginate to encapsulate G4. Initially, the beads were not well formed until a maximum concentration of bovine gelatin (13% w/v) and genipin (50 mM) were used, respectively. Further screening of sodium alginate (from 1 to 5% w/v) was done and resulted in well formation of beads, which indicated that the interaction degree of bovine gelatin was affected by the concentrations of genipin and sodium alginate.

Analysis of encapsulation yield (%) and bead strength (g) is shown in Table 2. The bead strength was examined before and after being exposed to SGF and SIF. It was demonstrated that the increase of sodium alginate from 1 to 5% (w/v) increased the EY% and the highest EY% (57.7%) was observed at 5% (w/v) of sodium alginate used. However, this value has no significant difference (*p* > 0.05) with 3 and 4% (w/v) of sodium alginate application. The bead strength (g) before and after being exposed to SGF and SIF also increased with the increase of sodium alginate used. In this study, the beads were targeted to be in strong condition before and after being exposed to SGF to ensure the encapsulated G4 was well protected and its viability remained. As approaching the intestinal region, beads were aimed to be weak so that the cells began to release into the area to boost the health effects.

The commonly used gelatin source in probiotic encapsulation process study is porcine gelatin. Based on the previous study by Annan et al. [16], the encapsulation of *B. lactis* Bb-12 was successfully obtained when porcine gelatin of 300 bloom strength was used at 10% (w/v) with the combination of 1% (w/v) sodium alginate and 10 mM of genipin. However, the study was limited to emulsion technique during encapsulation process and the study focused on the microsphere sizes, encapsulation yield, and disintegration time in SGF. This initiated our interest to use this formulation to extend our study on the encapsulation yield after extrusion technique has been applied, as well as bead strength before and after SGF and SIF exposures. Based on the author's previous study, using fish gelatin as one of the encapsulating matrices of probiotic gave a promising response [19]. As a result, in this study, further exploration using bovine gelatin with genipin and alginate as encapsulating matrices for G4 was conducted. To ensure the ability of bovine gelatin as encapsulating matrices, encapsulation yield data were obtained and compared with encapsulation yield of G4 using porcine gelatin following compositions suggested by Annan et al. [16] (Table 2).

The encapsulation yield of G4 using porcine gelatin was obtained at 45.9% ± 3.16, and this value was not significantly different with the findings in Table 2 when 1 and 2% (w/v) sodium alginate with the combination of bovine gelatin and genipin were used. Meanwhile, for bead strength, the value obtained was within the range when 2 and 3% (w/v) of sodium alginate were used. On the other hand, the bead strength after SIF exposure was recorded high when porcine gelatin/genipin/sodium alginate was used as compared to bovine gelatin/genipin/sodium alginate (Table 2). This provided an indication that using bovine gelatin with the combination of genipin and sodium alginate at suitable composition would yield similar performance as porcine gelatin matrices in encapsulation yield ability and to protect the cells during SGF exposure. However, it is interesting to note that the beads became weaker after SIF exposure when bovine gelatin was used as compared to the beads from porcine gelatin matrices. This suggested that using bovine gelatin matrices in encapsulating G4 has resulted in releasing more cells in this region.

Bovine gelatin produced from alkaline treatment that is known as type B gelatin has low isoelectric point ranging between pH 4.8 and 5.0 as reported by Aewsiri et al. [22]. The amphoteric characteristic of gelatin, which forms positive charge in low pH environment and changes to negative charge as pH environment changes beyond its isoelectric point, gave an opportunity in the combination between sodium alginate and gelatin. The interaction activity among the matrices occurred when alginate, which was natural amnionic polysaccharides, bound to gelatin polymer through negative-positive charges attraction. It was reported that as pH is lowered below the pKa values (ranging from 3.6 to 3.7) of alginates residues (D-mannuronic and L-guluronic acid), alginate was converted to alginic acid with the release of calcium ions [23] and with the presence of positive charge from gelatin polymer. The interaction became more stable, and this might be the reason for the bead strength to be stronger after SGF exposure with low pH condition (pH 1.2) introduced in this study. In contrast, after the beads were exposed to SIF with pH 6.5 for 3 h, the process of interaction was believed to be weak. This was due to the positive charge of bovine gelatin polymer changed to negative charge as the pH introduced was beyond its isoelectric point. As this occurred, negative charge of both matrices started to repel each other and caused bead strength to reduce. As compared to porcine gelatin, the isoelectric point was reported ranging from pH 6 to pH 9 [24] and the exposure of those beads in pH 6.8 environment resulted in a little influence on the interaction degradation process with alginate.

The role of genipin as a cross-linking agent to stabilise the gelatin was reported by Jin et al.[25] and Annan et al. [16]. A cross-linking activity between gelatin and genipin produced insoluble networks of gelatin with bond between amino acid residues through intrastrand, intramolecular, or intermolecular cross-link, leading to desirable increase in mechanical and thermal stabilities [26]. It is also reported to improve the stability of the beads in the simulated human gastrointestinal environment [27].

In this present study, the combination of bovine gelatin, genipin, and sodium alginate as encapsulating matrices was believed to have great potential as porcine gelatin application. As a result, further optimisation was carried out to obtain the optimum bovine gelatin, genipin, and sodium alginate concentrations for the better desired responses. The range of those encapsulating matrices was determined based on the screening experiment, and it is shown in Table 1.

### 3.2. Optimisation of Encapsulating Matrices Using Face-Centered Composite Design (FCCD)

The results for the bead strength of encapsulated G4 before SGF (*y*_1_) and after SGF (*y*_2_), SIF (*y*_3_), and encapsulation yield (*y*_4_) are presented in Table 3. The experimental design was a face-centered full factorial design (FCCD) with three factors (bovine gelatin, genipin, and sodium alginate) and three levels (−1, 0, and +1) with five replicates at the design centre. Centre points with all factors facilitated in understanding the curvature, and the replication helped to estimate pure error [28]. The methodology has been successfully employed in several optimisation studies for multiple regression analysis data obtained from the designed experiments to simultaneously solve multivariate equations [27–29]. As for the model selection, high *R*^2^ value or coefficient of determination is selected. *R*^2^ is defined as the ratio of explained variation to the total variation and is a measure of degree of fit. Besides, model analysis using analysis of variance (ANOVA) and the “lack of fit” test was also used for selection of adequate models [30].

#### 3.2.1. Bead Strength before SGF Exposure (*y*_1_)

**Figure 1:**
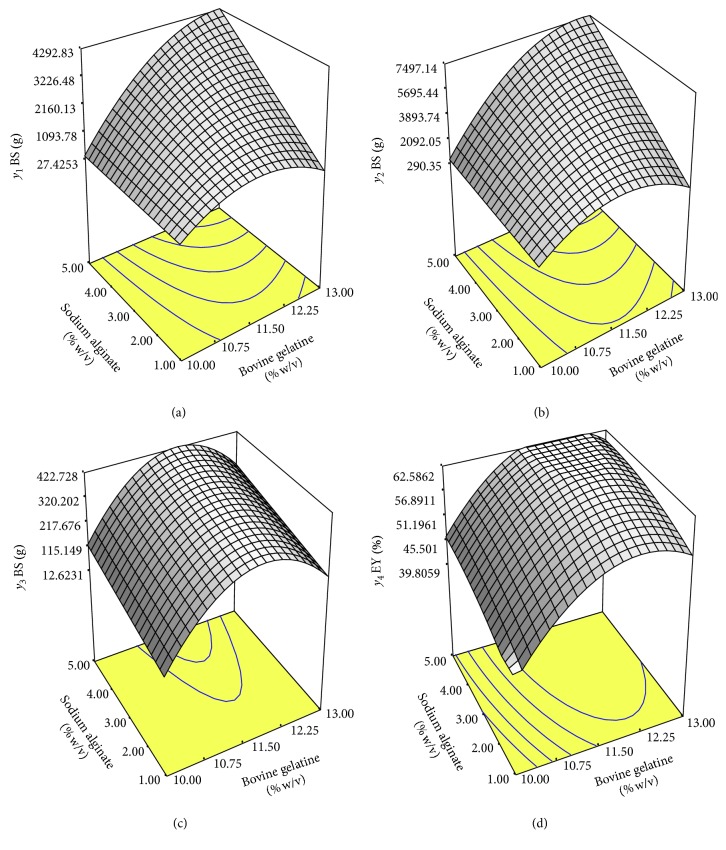
Response surface plot for (a) *y*_1_: bead strength (BS) before SGF exposure, (b) *y*_2_: bead strength (BS) after SGF exposure, (c) *y*_3_: bead strength (BS) after SIF exposure, and (d) *y*_4_: encapsulation yield from the quadratic mathematical model (genipin was set at 30 mM).

Based on the coefficient estimate of the experimental result, the following second-order equation explained the *y*_1_ response developed (Table 4). Referring to the equation, bovine gelatin and sodium alginate presented a big influence on the bead strength (*y*_1_). However, the addition of genipin was also important to improve the response. The significance of these factors on the bead strength can be determined by referring to ANOVA and regression analysis shown in Table 4. Highly significant factors on the response (*p* < 0.0001) were observed with bovine gelatin and sodium alginate. Meanwhile, the use of genipin resulted in a *p* value of 0.0438, which also remained significant to the response. The ANOVA result of this quadratic model was significant with *p* value for the bead strength (*y*_1_) lower than 0.05 (*p* < 0.0001) while the coefficient determination (*R*^2^) was found to be near to 1 (*R*^2^ = 0.9645), indicating the high correlation between the experimental and predicted value. The *p* value for “lack of fit” test of the response was higher than 0.05 (*p*=0.1226), indicating that the value for the model was insignificant and acceptable for the process. Adequate precision measured the signal to noise ratio, and the ratio greater than four was desirable. The adequate precision for this response (*y*_1_) was 25.987, and this high value demonstrated that the model was significant for the optimisation process. The relationship between the factors and the response (*y*_1_) was determined by plotting three-dimensional (3D) curves. By fixing the genipin level of 30 mM, the combined effect on bovine gelatin and sodium alginate on bead strength is illustrated in Figure 1(a). It can be noted that the *y*_1_ response value increased in accordance with the high level of bovine gelatin from 10 to 13% (w/v), when sodium alginate was maintained at 5% (w/v). In contrast, when sodium alginate was used at 1% (w/v), the bead strength increased only until bovine gelatin was increased to 11.50% (w/v) and started to decline when bovine gelatin concentrations continued to increase until 13% (w/v). Sodium alginate concentrations seemed to produce only minor influence on bead strength values for the low level of bovine gelatin (10% w/v), while for the highest concentrations of bovine gelatin (13% w/v), sodium alginate concentrations changes led to great effect on the response (*y*_1_). Our findings revealed that the higher levels of sodium alginate and bovine gelatin with the suitable genipin level could indeed form better bead strength (g), indicating that interaction charges between bovine gelatin and alginate polymers as well as intermolecular cross-links between amino acids residues of bovine gelatin and genipin were sufficient to form a stable binding activity. In this study, the bead strength was desired to be high so that it reflected that the probiotic G4 was well protected in the beads and was able to maintain the cell viability prior to SGF and SIF treatments.

#### 3.2.2. Bead Strength after SGF Exposure (*y*_2_)

The bead strength was determined after being exposed to SGF for 2 h and referred as *y*_2_ response (Table 4). A quadratic model was selected based on the highest-order polynomial with significant (*p* < 0.05) coefficients. The regression model equation after ANOVA for *y*_2_ response to the change of independent variables can be predicted by the equation shown in Table 4. Based on the equation, bovine gelatin (*x*_1_) and sodium alginate (*x*_3_) presented important factors affecting the strength of the beads after SGF exposure while genipin (*x*_2_) was shown to have little effect on the response.

ANOVA and regression analysis for *y*_2_ response are explained in Table 4. The independent variables, namely, *x*_1_, *x*_3_, *x*_1_^2^, and *x*_1_*x*_3_ showed high significance at the level *p* < 0.0001 while *x*_2_ remained significant (*p*=0.0165) on the response although the *p* value was slightly high compared to other variables. The model *F* value of 52.78 and *p* value of less than 0.05 implied that the model was significant. Other criteria such as “lack of fit” was not significant relative to pure error, and this insignificant “lack of fit” was good [31]. The coefficient determination (*R*^2^) calculated was 0.9531, suggesting that the model could explain 95.31% of the variability. Adequate precision showed ratio greater than 4, suggesting that the model showed adequate signal to noise ratio and it was acceptable. The model was applied to obtain optimal conditions of encapsulating matrices for optimum bead strength after SGF exposure (*y*_2_) by plotting 3D graphical methodology as shown in Figure 1(b). The plot was similar to the response surface plot for *y*_1_ in Figure 1(a). The highest bead strength after SGF exposure (*y*_2_) was noticed when bovine gelatin and alginate were at the highest concentrations, respectively. The lowest *y*_2_ response can be observed at the lowest encapsulating matrices used with 10% (w/v) of bovine gelatin and 1% (w/v) of sodium alginate combination. In this plot, the concentration of genipin was fixed to 30 mM. In this study, the bead strength was targeted to be high to protect the cells from being released into the gastric region. High viability of the cells is desired to remain in this region to ensure adequate densities are able to reach at the target site in the intestine and hence provide the host with a beneficial health effect.

#### 3.2.3. Bead Strength after SIF Exposure (*y*_3_)

After 3 h being exposed to SIF (pH 6.5), the bead strength was determined, and it is presented in Table 4. The bead strength varied with the range between 66.21 and 476.09 g. The values indicated that the response (*y*_3_) depended on the level of bovine gelatin, genipin, and sodium alginate used. Using ANOVA, the equation was generated (Table 4). Referring to Table 4, all independent variables (*x*_1_, *x*_2_, *x*_3_, *x*_1_^2^, *x*_2_^2^, and *x*_2_*x*_3_) shown were significant with a *p* value below 0.05. The experimental results were modelled using the quadratic model according to their significant *p* value (*p* < 0.0001) and insignificant “lack of fit” tests (*p*=0.5770). The model with no significant “lack of fit” was appropriate for the description of the response surface [32]. High values in coefficient determination (*R*^2^ = 0.9509) and adequate precision (19.25) demonstrated that the model selected was significant for the optimisation procedure. To understand the interaction of factors on the *y*_3_ response, the curve of response surface dimension was plotted as shown in Figure 1(c). By fixing genipin at 30 mM, it was noted that using bovine gelatin at 11.50% (w/v) with the increase of sodium alginate, a high value of bead strength after SIF exposure can be predicted. Meanwhile, the low value of bead strength after SIF treatment can be expected when bovine gelatin and sodium alginate were at minimum concentrations with 10 and 1% (w/v), respectively. However, the bead strength increased as sodium alginate concentration increased. Similar pattern was also observed when bovine gelatin was used at 13% (w/v). This finding revealed that the concentrations of sodium alginate were able to influence the bead strength after SIF exposure, suggesting that as sodium alginate concentration increased in the system, the bead structure density increased. It was suggested by Mandal et al. [33] that the use of high concentration sodium alginate of up to 4% (w/v) and above might cause the decrease in number and length of the pores, thus reducing the water molecule diffusion rate. Li et al. [14] also reported that the combination of sodium alginate and gelatin at high concentration reduced the percentage of water uptake and maintained their stability. In this study, low bead strength after SIF exposure was targeted to give indication that the relaxation of cross-linked activity between alginate-gelatin polymer-intermolecular genipin had occurred, suggesting that the cells started to be released into the intestinal region.

#### 3.2.4. EY% (*y*_4_)

The EY% (*y*_4_) was determined based on the experimental design as shown in Table 3. Generally, the encapsulation yield ranged between 35.2 and 65.7%. It was noted that the combination of bovine gelatin, genipin, and sodium alginate at the centre point presented better encapsulation yield compared to combinations of higher or lower concentrations. The experimental results were fitted to the quadratic model based on highest-order polynomial that presented the significant model (*p* < 0.05) and insignificant “lack of fit” tests. Model analysis and “lack of fit” tests were used for selection of the appropriate model, as outlined by Weng et al. [30] and Lee and Heo [34]. The equation in Table 4 was generated based on ANOVA. Based on the equation generated, the use of factor *x*_1_ (bovine gelatin) and *x*_3_ (sodium alginate) gave an impact to the EY% of G4 (*y*_4_). Factor *x*_2_ (genipin) showed a little influence on the response. The significant level of factors affecting EY% was detailed in ANOVA and regression analysis presented in Table 4. Obviously, the use of *x*_1_ and *x*_3_ was significant on the encapsulation yield as the *p* value was less than 0.05. Although *x*_2_ addition was not significant on the response, the use of this matrix remained important in the encapsulation process in this study due to its intermolecular cross-linking ability to bovine gelatin especially when the gelatin source used was low at bloom strength. The promising effect of cross-linking activity between genipin and gelatin with low bloom strength (<300 bs) was reported by Annan et al. [21] in *B. lactis* Bb-12 encapsulation study and by Chiono et al. [35] in biomedical applications. The model confidence was referred to the significant *p* value (*p* < 0.0001) and insignificant “lack of fit” tests (*p*=0.0959). The regression analysis showed that the coefficient of determination (*R*^2^) value was 0.8893, which was acceptable. The closer the *R*^2^ value to unity, the better the empirical model fitting the actual data, and the *R*^2^ value more than 0.85 was relatively adequate for prediction purpose [36]. The high value of adequate precision (more than 4) was also desirable, indicating an adequate signal for optimisation process. The response surface plots were generated to determine the interaction of all factors on *y*_4_ response. Interaction between bovine gelatin and sodium alginate on EY% of G4 is demonstrated in Figure 1(d). In this plot, genipin was set at 30 mM. It was noted that bovine gelatin was an important factor on the *y*_4_ response with the presence of sodium alginate. However, the use of bovine gelatin was limited between 11.50 and 12.3% (w/v). Further increase in the concentrations of gelatin, resulted in the reduction of encapsulation yield. This might be due to the increased of solution viscosity resulting in inefficient homogenisation process during mixing, thus leading to low entrapped cells. It is also possible that the entrapped cells were not released during encapsulation yield analysis when higher gelatin and sodium alginate concentrations were used due to strong adhesive activity [37] or the cells were injured during homogenisation due to the increase in stirring rate, thus leading to the decrease in the apparent encapsulation yield obtained [15]. Several researchers suggested that sodium alginate used ranging from 0.75 to 1.8% (w/v) [38], 1 to 3% (w/v) [32], 2 to 3% (w/v) [14], and 1 to 2% (w/v) [37] was able to present an optimum viscosifier ability that may be attributed to the bioadhesive property and facilitate the cell encapsulation process.

### 3.3. Verification

A numerical optimisation using the desired approach was employed to develop a new formulation of bovine gelatin, genipin, and sodium alginate with the desired responses. Constraints like maximising the bead strength before and after being exposed to SGF as well as encapsulation yield besides minimising the bead strength after SIF exposure were set as the goals to locate the optimum settings of independent variables in the new formulation.

The optimised formulation was developed using 11.21% (w/v) bovine gelatin, 13.96 mM genipin, and 2.60% (w/v) sodium alginate. It was verified for the observed responses as predicted by the mathematical model shown in Table 5. It can be observed that the experimental and predicted values were not significantly different (*p* > 0.05) for all the responses. The low value of error indicates high prognostic ability of the response surface methodology [39].

## 4. Conclusions

In conclusion, bovine gelatin source with the combination of genipin and sodium alginate can be successfully used as encapsulating matrices for G4. The bead strength performance before and after SGF and SIF treatments was comparable to porcine gelatin beads. High encapsulation yield also presented a promising characteristic of these potential encapsulating matrices. Further optimisation using FCCD-RSM revealed that bovine gelatin with the combination of genipin and sodium alginate with the suitable compositions influenced the desired responses. The developed models can be used for prediction of the amounts of these encapsulating matrices to obtain the optimum bead strength before and after SGF and SIF exposure as well as encapsulation yield. The models can also be used to identify the most important factors for the responses. During optimisation, RSM analysis showed that the optimum responses were obtained at 11.21% (w/v) of bovine gelatin, 13.96 mM of genipin, and 2.60% (w/v) of sodium alginate [40]. This optimised encapsulating matrix was able to form beads as strong as porcine gelatin beads before and after SGF exposure. However, the beads of optimised formulations became weaker than porcine gelatin beads after SIF treatment, indicating positive effects on G4 releasing activity into the target region. These findings can be concluded that the optimised bovine gelatin beads with genipin and sodium alginate combinations have a good potential to be used as a halal delivery vehicle for probiotic cells.

## Figures and Tables

**Table 1:** Independent variables (bovine gelatin, sodium alginate, and genipin) and their levels in the experimental design.

Independent variables	Code levels		
	−1	0	+1
Bovine gelatin (% w/v)	10	11.5	13
Sodium alginate (% w/v)	1	3	5
Genipin (mM)	10	30	50

**Table 2:** Encapsulation yield (%) of *B. pseudocatenulatum* G4 and bead strength (g) before and after exposed to simulated gastric fluid (SGF) and simulated intestinal fluid (SIF).

Sodium alginate (% w/v)	Bead strength (g)			Encapsulation yield (%)
	Before	After SGF	After SIF	
1^*∗*^	313.89 ± 6.14^a^	525.11 ± 5.52^a^	154.21 ± 4.40^a^	45.06 ± 1.83^a^
2	848.62 ± 4.75^b^	1031.09 ± 4.58^b^	520.92 ± 5.47^b^	45.72 ± 0.87^a^
3	3118.66 ± 11.02^c^	5912.28 ± 9.79^c^	484.75 ± 4.65^c^	54.22 ± 3.54^b^
4	3748.18 ± 15.41^d^	5575.34 ± 14.28^d^	627.83 ± 5.31^d^	57.53 ± 2.50^b^
5	4299.80 ± 17.49^e^	7214.19 ± 6.66^e^	749.25 ± 6.56^e^	57.66 ± 2.68^b^
^*∗∗*^	2477.55 ± 12.95^f^	5039.21 ± 24.42^f^	991.72 ± 9.28^f^	45.94 ± 3.16^a^

^*∗*^Bovine gelatin (13% w/v) and genipin (50 mM). ^*∗∗*^Formula as suggested by Annan et al. [21] using porcine gelatin. ^1^SGF, simulated gastric fluid. ^2^SIF, simulated intestinal fluid. ^3^Encapsulation yield (N/N_0_ × 100): *N* = number of viable entrapped cells and *N*_0_ = number of viable free cells loaded into the encapsulation matrix. Different letters within columns are significantly different (*p* < 0.05). Values represent mean ± standard deviation. Data obtained were based on the 30 beads sample for the analysis.

**Table 3:** Experimental design and results using face-centered full factorial design (FCCD): bovine gelatin, sodium alginate, and genipin.

Run	Bovine gelatin (% w/v), *x*_1_	Genipin (mM), *x*_2_	Sodium alginate (% w/v), *x*_3_	Responses^1^			
				BS^2^ before (g), *y*_1_	BS after SGF^3^ (g), *y*_2_	BS after SIF^4^ (g), *y*_3_	EY^5^ (%), *y*_4_
13	10	10	1	91.4	177.4	66.2	35.2
14	10	10	5	292.9	654.9	120.2	43.8
15	10	30	3	178.1	269.4	75.1	41.6
16	10	50	1	154.7	249.0	102.2	39.6
12	10	50	5	532.9	1025.2	228.0	44.9
3	11.5	10	3	1860.0	3797.4	411.0	54.4
1	11.5	30	1	1567.3	2727.4	309.1	50.1
2	11.5	30	3	2462.3	4604.6	380.1	62.4
6	11.5	30	3	2678.5	4551.7	372.1	65.3
11	11.5	30	3	2399.6	4469.3	298.0	61.0
7	11.5	30	3	2362.2	5288.1	382.1	62.2
19	11.5	30	3	2148.8	4463.9	375.1	60.1
18	11.5	30	5	2962.7	4928.0	417.1	65.7
10	11.5	50	3	2919.0	4199.3	420.1	61.6
8	13	10	1	336.5	396.9	199.1	55.4
17	13	10	5	4310.0	7703.5	249.0	61.5
9	13	30	3	3111.3	5415.1	206.1	58.1
5	13	50	1	349.6	542.9	260.4	52.2
4	13	50	5	4435.3	6975.5	476.1	54.3

^1^All factorial and axial points are means of duplicate; ^2^BS, bead strength; ^3^SGF, simulated gastric fluid; ^4^SIF, simulated intestinal fluid; ^5^EY, encapsulation yield.

**Table 4:** ANOVA and regression analysis for the responses of bead strength before (*y*_1_) and after SGF (*y*_2_)/SIF exposure (*y*_3_) as well as encapsulation yield (*y*_4_).

Source	Sum of squares	DF^1^	Mean square	*F* value	*p* value
*y*_1_2368.9 1129.27*x*_1_ 154.09*x*_2_ 1003.4*x*_3_ 989.7*x*_1_^2^ 934.9*x*_1_*x*_3_					
Model	3.5*E* + 007	5	6.938*E* + 006	70.7	<0.0001^a^
Residual	1.3*E* + 006	13	98088.27		
Lack of fit	1.1*E* + 006	9	1.255*E* + 005	3.5	0.1226
Pure error	1.5*E* + 005	4	36366.13		
Factor^b^/intercept					
*x*_1_	1.3*E* + 007	1	1.275*E* + 007	130.1	<0.0001
*x*_2_	2.4*E* + 005	1	2.374*E* + 005	2.4	0.0438
*x*_3_	1.0*E* + 007	1	1.007*E* + 007	102.6	<0.0001
*x*_1_^2^	4.6*E* + 006	1	4.639*E* + 006	47.3	<0.0001
*x*_1_ *x*_3_	6.9*E* + 002	1	6.993*E* + 002	71.2	<0.0001
*R* ^2^ = 96.45%, adequate precision = 25.99					

*y*_2_4336.6 1865.8*x*_1_ 26.8*x*_2_ 1719.5*x*_3_ 1995.7*x*_1_^2^ 1560.6*x*_1_*x*_3_					
Model	1.0*E* + 008	5	2.055*E* + 007	52.8	<0.0001^a^
Residual	5.1*E* + 008	13	3.893*E* + 005		
Lack of fit	4.6*E* + 006	9	5.086*E* + 005	4.2	0.0898
Pure error	4.8*E* + 005	4	1.207*E* + 005		
Factor^b^/intercept					
*x*_1_	3.5*E* + 007	1	3.5*E* + 007	89.4	<0.0001
*x*_2_	6852.4	1	6852.4	1.0	0.0165
*x*_3_	2.9*E* + 007	1	2.9*E* + 007	75.9	<0.0001
*x*_1_^2^	1.9*E* + 007	1	1.9*E* + 007	48.5	<0.0001
*x*_1_ *x*_3_	1.9*E* + 007	1	1.9*E* + 007	50.1	<0.0001
*R* ^2^ = 95.31%. Adequate precision = 20.6					

*y*_3_359.9 79.9*x* 44.1*x*_1_^2^ 55.3*x*_3_ 212.0*x*_1_^2^ 62.9*x*_2_^2^ 29.7*x*_2_*x*_3_					
Model	2.8*E* + 005	6	46605.7	30.7	<0.0001^a^
Residual	1444.0	12	1203.5		
Lack of fit	9346.4	8	1168.3	0.9	0.5770
Pure error	5095.7	4	1273.9		
Factor^b^/intercept					
*x*_1_	63854.5	1	63854.5	53.1	<0.0001
*x*_2_	19458.7	1	19458.7	16.2	0.0017
*x*_3_	30629.6	1	30629.6	25.2	0.0003
*x*_1_^2^	1.4*E* + 005	1	1.4*E* + 005	25.5	<0.0001
*x*_2_^2^	12503.9	1	12503.9	117.9	0.0073
*x*_2_ *x*_3_	7065.0	1	7065.0	5.9	0.0321
*R* ^2^ = 95.09%, adequate precision = 19.27					

*y*_4_60.3 7.7*x*_1_ 0.2*x*_2_ 3.8*x*_3_ 11.7*x*_1_^2^					
Model	1371.9	4	343.0	28.1	<0.0001
Residual	170.8	14	12.2		
Lack of fit	155.9	10	15.8	4.0	0.0959
Pure error	15.4	4	3.86		
Factor^b^/intercept					
*x*_1_	584.9	1	584.9	47.9	<0.0001
*x*_2_	0.5	1	0.5	0.1	0.8340
*x*_3_	141.3	1	141.3	11.6	0.0043
*x*_1_^2^	645.2	1	645.2	52.8	<0.0001
*R* ^2^ = 88.93%, adequate precision = 15.11					

^a^Significant at *α* = 0.05. ^1^DF, degree of freedom; *x*_1_, bovine gelatin (% w/v); *x*_2_, genipin (mM); *x*_3_, sodium alginate (% w/v).

**Table 5:** Comparison of experimental and predicted bead strength and encapsulation yield using optimised bovine gelatin, genipin, and sodium alginate.

	BS (g)^1^			EY (%)^2^
	Before SGF	After SGF	After SIF	
Experimental	1821.33 ± 10.02^a^^*∗*^	3594.33 ± 5.69^a^	339.55 ± 12.67^a^	56.28 ± 2.29^a^
Predicted	1826.15^a^	3597.91^a^	335.37^a^	57.46^a^
Error (%)	0.26	0.07	1.28	2.10

^1^BS, bead strength; ^2^EY, encapsulation yield. ^*∗*^Values in the same column with different letters were significantly different (*p* < 0.05). Values denote mean ± standard deviation.

## Data Availability

The data used to support the findings of this study are available from the corresponding author upon request.
